# Effects of saliva collection using cotton swabs on melatonin enzyme immunoassay

**DOI:** 10.1186/1740-3391-9-1

**Published:** 2011-01-10

**Authors:** Tomoaki Kozaki, Soomin Lee, Takayuki Nishimura, Tetsuo Katsuura, Akira Yasukouchi

**Affiliations:** 1 National Institute of Occupational Safety and Health, 6-21-1 Nagao, Tama-ku, Kawasaki 214-8585, Japan; 2 Faculty of Engineering, Chiba University, 1-33 Yayoi-cho, Inage-ku, Chiba 263-8522, Japan; 3 Graduate school of Design, Kyushu University, 4-9-1 Shiobaru, Minami-ku, Fukuoka, Japan; 4 Faculty of Design, Kyushu University, 4-9-1 Shiobaru, Minami-ku, Fukuoka, Japan

## Abstract

**Background:**

Although various acceptable and easy-to-use devices have been used for saliva collection, cotton swabs are among the most common ones. Previous studies reported that cotton swabs yield a lower level of melatonin detection. However, this statistical method is not adequate for detecting an agreement between cotton saliva collection and passive saliva collection, and a test for bias is needed. Furthermore, the effects of cotton swabs have not been examined at lower melatonin level, a level at which melatonin is used for assessment of circadian rhythms, namely dim light melatonin onset (DLMO). In the present study, we estimated the effect of cotton swabs on the results of salivary melatonin assay using the Bland-Altman plot at lower level.

**Methods:**

Nine healthy males were recruited and each provided four saliva samples on a single day to yield a total of 36 samples. Saliva samples were directly collected in plastic tubes using plastic straws, and subsequently pipetted onto cotton swabs (cotton saliva collection) and into clear sterile tubes (passive saliva collection). The melatonin levels were analyzed in duplicate using commercially available ELISA kits.

**Results:**

The mean melatonin concentration in cotton saliva collection samples was significantly lower than that in passive saliva collection samples at higher melatonin level (>6 pg/mL). The Bland-Altman plot indicated that cotton swabs causes relative and proportional biases in the assay results. For lower melatonin level (<6 pg/mL), although the BA plots didn't show proportional and relative biases, there was no significant correlation between passive and cotton saliva collection samples.

**Conclusion:**

Our findings indicate an interference effect of cotton swabs on the assay result of salivary melatonin at lower melatonin level. Cotton-based collection devices might, thus, not be suitable for assessment of DLMO.

## Background

Melatonin, produced by the pineal gland [1,2], has often been assessed for determination of human circadian phase as dim light melatonin onset (DLMO) [3-6]. Assessing salivary melatonin has recently been used as an alternative method for blood analysis because the level of salivary melatonin is correlated with that of blood melatonin [7]. Furthermore, collecting salivary samples is less intrusive and easier for participants than collecting urine and blood samples.

Although various acceptable and easy-to-use devices have been used for saliva collection, cotton swabs are among the most common. However, previous studies have reported that cotton swabs yield low levels of melatonin [8,9]. In these studies, saliva samples were collected by spitting into a clear bottle (passive saliva collection), and exogenous melatonin was artificially added to the samples and loaded on cotton swabs (cotton saliva collection). Thus, the cotton swabs were not placed into the mouth; those studies were defined as '*in vitro*' experiments. In contrast, Weber et al. [8] collected saliva samples using cotton swabs placed into the mouth ('*in vivo*' experiment) and examined the effect on the melatonin assay result. They demonstrated a reducing effect of the cotton swabs on the salivary melatonin assay result. They suggested that the difference between the '*in vitro*' and '*in vivo*' experiments may be due to the presence of high molecular-weight proteins (mucins), which may cover the cotton swabs and prevent binding of melatonin to the cotton swabs. However, our previous '*in vitro*' study indicated subject-specific variability in the effect of cotton swabs on salivary cortisol assay results [10]. This finding implies variability in the presence of mucins between saliva samples; some saliva samples may contain a small amount of mucins. Thus, an '*in vitro*' experiment is appropriate for demonstrating the effect of cotton swabs.

Weber et al. [8] examined the effect of cotton swabs on 'natural' endogenous melatonin as an '*in vitro*' experiment. However, the effect was demonstrated at a higher level (>9.8 pg/mL), whereas DLMO thresholds of many studies were lower than 6 pg/mL [11-14]. Weber et al. [8] also indicated that the recovery rates of exogenous and endogenous melatonin from cotton swabs were different. Furthermore, earlier studies [8,9] have estimated melatonin recovery from cotton swabs. This statistical method is not adequate for detecting an agreement between the two measurement methods, i.e. cotton saliva collection and passive saliva collection, hence a test for bias is needed [9]. In the present '*in vitro*' study, we estimated the effect of cotton swabs on the lower level (<6 pg/mL) of salivary endogenous melatonin assay results, the agreement between collection methods and the bias caused by cotton swabs.

## Methods

### Subjects

Nine healthy males (age, 20-31 years) were included after obtaining written consent. The subjects had no medical conditions that would interfere with the results. All subjects were non-smokers and were instructed to abstain from alcohol for 1 day as well as from caffeine, food and brushing their teeth for 2 h before the samples were collected.

### Saliva sample collection

Each subject provided four saliva samples at night (2200 h to 0100 h) for a total of 36 samples. The saliva samples were collected under dim conditions (<30 lx) because melatonin secretion is acutely suppressed by bright light [15]. As an '*in vitro*' study, saliva samples were directly collected in clear sterile plastic tubes using sterile plastic straws. A 1-mL aliquot of each saliva sample was pipetted onto a Salivette^® ^cotton swab (Sarstedt K. K., Tokyo, Japan) (cotton saliva collection) and into clear sterile plastic tubes (passive saliva collection). All saliva samples were centrifuged at 1500× *g *for 5 min at room temperature and then frozen at -30°C until being assayed.

### Salivary melatonin assay

The melatonin levels were analysed in duplicate using commercially available ELISA kits (Direct Saliva Melatonin ELISA; Bühlmann Laboratories, Allschwil, Switzerland), and the mean values of the duplicates were used for analysing the results. The kit sensitivity was 0.5 pg/mL. The intra- and inter-assay coefficients of variation were 12.6% and 22.9%, respectively.

### Statistics

The mean salivary melatonin levels were compared using a two-tailed paired *t*-test. Pearson's correlation coefficients were calculated between the passive saliva collection and cotton saliva collection samples. Bland-Altman (BA) plots [16] were used to detect agreement and bias. Statistical analyses were performed using SPSS version 16.0 (SPSS, Chicago, IL, USA). A p value <0.05 was considered statistically significant.

## Results

**Figure 1:**
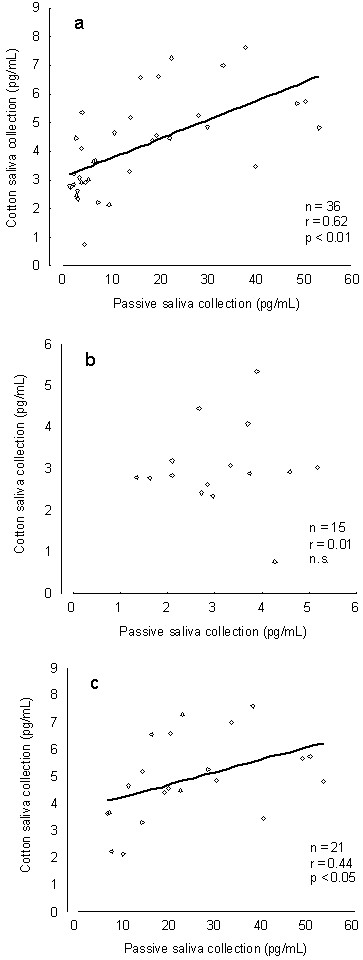
**Scatter plots of melatonin concentrations between passive and cotton saliva collection for all (a), lower-level (b), and higher-level (c) samples**.

Table 1 shows the mean and standard deviation of the melatonin concentrations and Pearson's correlation coefficient (r) for the lower (<6 pg/mL) and higher (>6 pg/mL) melatonin levels. The mean melatonin concentrations of all samples were significantly different between the passive and cotton saliva collections. The correlation between collection methods was significant (Figure 1a). No significant difference was observed for the mean lower-level melatonin concentrations between the cotton and passive saliva collections. Cotton saliva collection samples were not significantly correlated with passive saliva collection samples (Figure 1b). Although the mean higher-level melatonin concentration from the cotton saliva collection was significantly lower than that from passive saliva collection, a significant correlation was observed between the collection methods (Figure 1c).

**Table 1:** Pearson's correlation coefficient (r) and concentrations of passive saliva (P) and cotton saliva (C) melatonin for all, lower-level (<6 pg/mL), and higher-level (>6 pg/mL) samples.

	Mean and standard deviation (SD)		r
		
	P (pg/mL)	C (pg/mL)	P vs. C
<6 pg/mL	3.14 (1.10)	3.04 (1.04)	0.01
>6 pg/mL	24.22 (14.82)	4.91 (1.55)**	0.44*
All	15.43 (15.40)	4.13 (1.63)**	0.62**

**p < 0.01

**Figure 2:**
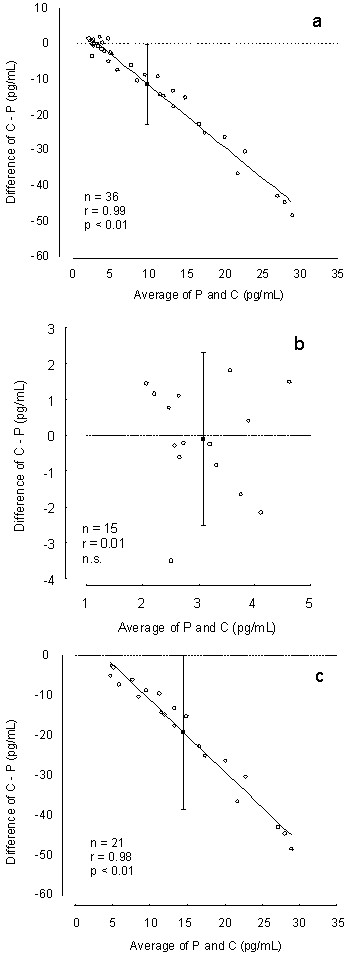
**BA plots of passive and cotton saliva collection for all (a), lower-level (b), and higher-level (c) samples**.

The 95% confidence intervals (CIs) for the difference between cotton and passive saliva collection samples (the difference of C-P) was not zero in the BA plots of all samples, indicating a relative bias caused by the cotton swabs (Figure 2a); the CI extended from -0.0001 to -22.598. A proportional bias caused by cotton swabs was indicated because the average and the C-P difference from the BA plots were significantly correlated. For the lower level (Figure 2b), the CI extended from 2.323 to -2.5708, and no significant correlation between the average and the C-P difference was observed. No relative or proportional biases were observed for the lower level. The CI for the higher level extended from -0.001 to -38.606, and a significant correlation was observed between the average and the C-P difference (Figure 2c). Thus, the higher-level BA plots indicated relative and proportional biases.

## Discussion

A significantly low melatonin concentration was obtained from the cotton saliva collection in all samples compared with that from passive saliva collection, and the decreasing rate was 26.8%. This finding is in accordance with the results of earlier studies [8,9]. In addition, the BA plots indicated a relative bias. Although cotton saliva collection was significantly correlated with passive saliva collection, the BA plots indicated that the cotton swabs introduced a proportional bias. The average of P and C was negatively correlated with the differences in C-P (Figure 2), and the correlation coefficient was very high (r = 0.99). The higher-level samples showed similar findings for all samples, indicating that cotton swabs absorb melatonin molecules in proportion to the higher melatonin concentration (>6 pg/mL).

For the lower melatonin level (<6 pg/mL), although the BA plots did not show proportional and relative biases, no significant correlation was observed between passive and cotton saliva collection samples. These findings indicate that cotton swabs caused a depression and an elevation in the assay results. Some substances contained in the cotton may non-specifically link or cross-link with the specific antibody used for the assay [17]. Although no evidence exists, a cotton-induced non-specific linking and/or cross linking may have slightly elevated the assay result, causing an interference effect of the cotton swabs for the lower melatonin level.

The '*in vivo*' experiment by Weber et al. [8] demonstrated a low effect of cotton swabs at higher melatonin levels (>9.8 pg/mL). They argued that high molecular-weight proteins (mucins) in saliva may have prevented the binding of melatonin to cotton. However, our present lower melatonin level (<6 pg/mL) findings indicate that cotton swabs may elevate the assay result. Thus, cotton-based collection devices may be inappropriate for assessing DLMO.

The present findings indicate the effects of collecting saliva on cotton swabs on assay results. In particular, cotton swabs did not result in a depressed assay result for the lower melatonin level, whereas lower concentrations were obtained on cotton swabs with the higher melatonin level, as in earlier studies [8,9]. In contrast, these devices are useful for saliva collection because they can provide pure samples. Thus, non-cotton-based devices such as polyester are recommended [8].

## Competing interests

The authors declare that they have no competing interests.

## Authors' contributions

All authors participated in design, acquisition of data, analysis and interpretation of data, and manuscript preparation. They approved the manuscript.
